# Permanent Central Diabetes Insipidus with Complete Regression of Pituitary Stalk Enlargement After 4 Years of Follow−up

**DOI:** 10.4008/jcrpe.v1i1.4

**Published:** 2008-08-06

**Authors:** Gönül Öcal, Zeynep Şıklar, Merih Berberoğlu, Pelin Bilir, Özlem Engiz, Suat Fitoz, Serap Arıcı

**Affiliations:** 1 Ankara University, Faculty of Medicine, Pediatric Endocrinology Unit, Ankara, Turkey; 2 Ankara University, Faculty of Medicine, Department of Radiology, Ankara, Turkey; +90−312 595 67 91+90−312 319 14 40zeynepsklr@gmail.comAnkara Üniversitesi Tıp Fakültesi Pediatrik Endokrinoloji Bilim Dalı Cebeci− Ankara/Turkey

**Keywords:** Pituitary stalk thickness, central diabetes insipidus, multiple anterior pituitary hormone deficiency

## Abstract

A 14 year−old patient was admitted because of a history of polyuria and polydipsia. A diagnosis of central diabetes insipidus (CDI) accompanied by growth hormone (GH) and gonadotropin deficiency was made. Hypophyseal magnetic resonance imaging (MRI) of the patient demonstrated isolated pituitary stalk enlargement. Although GH deficiency and gonadotropin deficiency were transient, CDI was persistent despite the regression of the pituitary stalk enlargement over the 4 years of follow−up.

**Conflict of interest:**None declared.

## INTRODUCTION

Central diabetes insipidus (CDI) is a condition characterized by excretion of abnormally large volumes of dilute urine due to deficiency of the arginine−vasopressin peptide.(1, 2, 3, 4) CDI may result from a lesion of the hypothalamic neurohypo− physeal system. Magnetic resonance imaging (MRI) has revealed loss of bright T1 signal of the posterior pituitary with or without pituitary stalk thickening (PST) in some cases of idiopathic CDI as well as some cases of secondary CDI due to an infiltrative process or neoplasia. The clinical features of a lesion involving the hypothalamus and pituitary stalk depend on its location, its degree of extension, and rate of its progression.(5, 6, 7, 8) Pituitary stalk involvement may lead to isolated or multiple anterior pituitary hormone dysfunction and/or posterior pituitary dysfunction. There may be a change in PST at follow up.(9)

This paper presents the case of a 14 year−old boy with transient pituitary stalk enlargement and growth hormone (GH) deficiency and central hypogonadism, and with a persistent loss of MRI − bright T1 signal of posterior pituitary and CDI over a four−year follow up period.

## CASE REPORTS

A 14 year−old male patient with a history of polyuria and polydipsia in the past 2 months was admitted to our hospital. He was prepubertal and of short stature (height SDS: −2.26, parentally adjusted height SDS: −2.64). He ingested 8 L of fluid and urinated 5−6 L daily. At initial evaluation he appeared well hydrated and had normal vital signs. His basal urinary osmolality, serum osmolality and the ratio of urinary osmolality to serum osmolality were, 40 mosm/kg H_2_O, 299 mosm/kg H_2_O and 0.13, respectively. Serum sodium, potassium, calcium, phosphorus levels were within normal limits. Water deprivation test and desmopressin (dDAVP) test confirmed the diagnosis of complete CDI. After subcutaneous dDAVP administration, urinary osmolality increase from the baseline was 174%. There was no family history of CDI, no history of head trauma/injury or evidence of systemic diseases such as Langerhans cell hystiocytosis (LCH), sarcoidosis, tuberculosis or neurological deficits.

MRI of the pituitary region showed a thickened pituitary stalk (5 mm) and loss of the hyperintense signal of the posterior pituitary. Transsphenoidal biopsy of the lesion was not done. Desmopressin treatment was initiated. Complete anterior pituitary endocrine function evaluation included measurement of growth hormone response to pharmacological stimulation tests (L−Dopa test and insulin tolerance test−ITT); baseline and ITT stimulated cortisol levels; thyroid function tests; basal testosterone level, luteinizing hormone (LH) and follicle stimulating hormone (FSH) response to gonadotropin releasing hormone (GnRH) stimulation, and prolactin level. The patient did not have central hypothyroidism, adrenocorticotropic hormone (ACTH) deficiency or hyperprolactinemia. He suffered from both complete growth hormone (GH) deficiency (L−Dopa stimulated GH peak was 0.65 ng/mL, ITT stimulated GH peak was 0.38 ng/mL) and hypogonadotropic hypogonadism. At 14 years of age he had bilateral testicular volumes of less than 4 mL, basal testosterone level was within the prepubertal range (13 ng/dL) and GnRH stimulated LH and FSH were 3.38 mIU/mL and 4.22 mIU/mL, respectively. GH stimulation tests were performed after priming with sex steroid. In the differential diagnosis, pituitary tumor (such as germinoma), LCH, sarcoidosis or tuberculosis were considered. High resolution CT of the thorax and a bone survey for LCH were normal. A negative result was obtained for human chorionic gonadotropin (hCG) and a−fetoprotein levels in the cerebrospinal fluid, which were measured to eliminate a diagnosis of germinoma. The patient had no systemic evidence of sarcoidosis, and hyperprolactinemia was not detected. Serum angiotensin converting enzyme was within normal levels. Chest Xray, intradermal tuberculin test, cerebrospinal fluid analysis yielded negative results for tuberculosis.

The patient was followed clinically, backed up with endocrine and MRI evaluation for 4 years, with no treatment other than desmopressin as a hormone substitution therapy.

**Figure 1:**
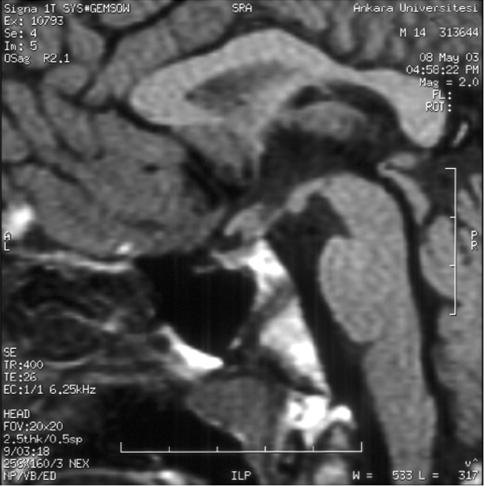
MRI at admission: A 5 mm infundibular thickness, absence of posterior hypophyseal bright spot

His pituitary stalk size showed a change over the four years of follow up. Hypophyseal MRI, which was taken every 3 to 4 months, demonstrated that PST enlarged from 5 mm to 7 mm during the first 10 months of follow−up. After that, complete regression of pituitary stalk enlargement occurred over the subsequent 14 months. The time interval between the diagnosis and the complete regression of PST enlargement was 2 years. PST decreased from 7 mm to 2.5 mm, (Figure 1, 2, 3) and retained its normal size for the last 2 years. The loss of bright signal on MRI remained unchanged during the follow−up period.

Despite improvement of the GH deficiency and hypogonadotropic hypogonadism, CDI was persistent. At the most recent evaluation when the patient was 17.9 years of age, GH response to ITT was 20 ng/mL, testicular volume was 20 mL and basal testosterone level was 232 ng/dL. His height was 169.8 cm, height SDS was −0.59, and he had a +1.67 SD height gain without GH treatment. Because of the regression of PST, absence of progression to any systemic disease or pituitary tumor over 4 years, an autoimmune hypophysitis was suspected in this patient.

**2:**
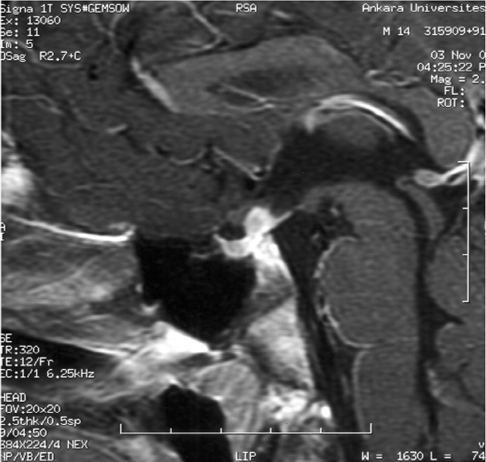
MRI at follow−up: Enlargement of PST from 5 mm to 7 mm

**3:**
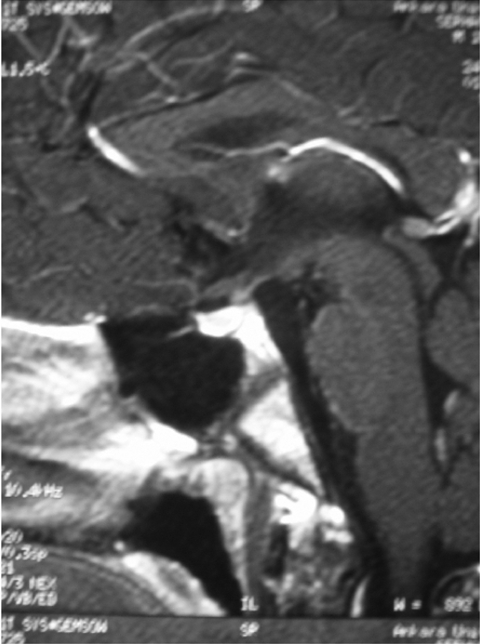
MRI at follow−up: Regression of the PST from 7 mm to 2.5 mm on MRI

## DISCUSSION

In this article clinical, hormonal and radiological features in a 14 years−old boy with persistent CDI but with regression of PST over the 4 years follow−up period is reported.

Mass lesions involving the hypothalamicpituitary system constitute diagnostic challenges for endocrinologists, particularly since the introduction of MRI. Isolated GH deficiency or multiple pituitary hormone deficiency (MPHD) develop in 90−94% of patients with CDI and PST.(4) Our patient presented with a combination of CDI and transient MPHD including GH deficiency and hypogonadotropic hypogonadism.

Pituitary stalk and hypothalamus involvement may be due to cystic, neoplastic, infectious and inflammatory processes.(3, 8, 9, 10, 11, 12, 13, 14, 15, 16, 17, 18, 19) PST has a wide differential diagnosis, but almost all patients present with CDI.(11) In one third of patients with CDI, the pituitary stalk is thickened, suggesting infiltrative disease.(4) Investigation for hypophyseal tuberculoma, sarcoidosis, LCH, germinoma and autoimmune hypophysitisis are important in the differential diagnosis of the condition.(10) In about 60% of patients with PST, the etiology of the CDI remains undetermined, but in approximately 15−20%, LCH and occult germinoma each account for the CDI. In the report by Czernichow et al(7) on 26 children with CDI and PST, four had germinoma, five had LCH, and 17 were diagnosed as idiopathic.

PST is defined as exceeding 2.5 mm in width.(9) The initial PST in our patient was 5 mm, and progressed to 7 mm within a 10 month period. This was followed by a spontaneous regression to 2.5 mm over the following 14 months, and retaining a normal size in the subsequent 2 years. Excluding the likely diseases in the differential diagnosis, the patient was initially designated as idiopathic, but progressive PST in the following 10 months of follow−up suggested an occult germinoma. However, normal cerebrospinal hCG and a−fetoprotein values and spontaneous regression of the PST over the subsequent 2 years without recurrence by the 4^th^ year, excluded this diagnosis. Imura et al(19) showed that autoimmune CDI could be due to lymphocytic infundibulo−neurohypophysitis proven by infiltration of lymphocytes and plasma−cells on biopsy samples of the pituitary stalk or suggested by presence of PST on MRI which disappeared over time spontaneously, just like in our patient. Although antibodies to AVP secreting cells have not been studied in our patient, the persistence of CDI despite improvement of the GH deficiency and hypogonadotropic hypogonadism suggest autoimmune hypophysitis.

In this patient, the pubertal delay could be attributed to constitutional pubertal delay. However, onset of puberty spontaneously following PST regression, together with improvement of GH deficiency, led us to think that the pubertal delay was due to transient hypogonadotropic hypogonadism. GH deficiency could also be attributed to false positive results of GH tests especially in peripubertal ages, but the tests had been conducted after priming with sex steroids and GH tests are still considered as the gold standard for the diagnosis of GH deficiency.

In conclusion, long term follow up should be made in children with unexplained CDI and PST. PST related endocrine disturbances such as GH deficiency, and hypogonadotrophic hypogonadism, with the exception of CDI, may be transient during long term follow−up with regression of PST. Provided cerebrospinal fluid investigation results are normal, biopsy of the enlarged stalk may not be necessary, since spontaneous recovery may occur.
